# Prevalence of Adverse Childhood Experience Exposure by Disability Status

**DOI:** 10.1001/jamahealthforum.2024.4881

**Published:** 2025-01-10

**Authors:** Sophia Miryam Schüssler-Fiorenza Rose, David H. Rehkopf, Michael P. Snyder, George M. Slavich

**Affiliations:** 1 Department of Genetics, Stanford University School of Medicine, Stanford, California; 2 Department of Epidemiology and Population Health, Stanford University, Stanford, California; 3 Department of Psychiatry and Biobehavioral Sciences, University of California, Los Angeles

## Abstract

This cross-sectional study uses a population-based dataset to examine the prevalence of adverse childhood experiences in people with many different types of disability.

## Introduction

Adverse childhood experiences (ACEs), including early-life abuse and neglect, can lead to lasting biological changes^1^ that increase risk for chronic disease and premature mortality.^2^ Disproportionate ACE exposure can also drive health disparities. ACEs are more prevalent among socioeconomically disadvantaged groups. People with disabilities are a socioeconomically disadvantaged group that faces considerable health disparities^3^; however, the complex association between ACE exposure and disability remains poorly understood. Population-based studies on ACEs and disability have used a limited definition of disability^4^ that does not distinguish between types of disability, rendering the understanding of ACE exposure in people with disabilities incomplete. To address this gap, we used a population-based dataset to examine for the first time, to our knowledge, the prevalence of ACEs in people with many different types of disability.

## Methods

The Behavioral Risk Factor Surveillance System is a cross-sectional, population-based telephone survey administered to community-dwelling adults. We combined data from the 39 states, and Washington, DC, that administered the optional ACE module from 2019 to 2022. The disability questions (yes/no) ask about serious difficulty with mobility, self-care (bathing/showering), independent living, hearing, vision, and cognition (eTable 1 in Supplement 1). The ACE questions cover abuse and family dysfunction occurring before age 18 years (eTable 2 in Supplement 1). The survey procedures in SAS, version 9.4 (SAS Institute), accounted for the complex survey design, and survey weights that adjust for sampling and nonresponse bias were used in all analyses. For states that contributed multiple years of data, we adjusted the weights by the proportion of the state’s participants in that year’s dataset over the total for all years contributed. We performed logistic regression to adjust for age (continuous), sex, and self-reported race and ethnicity. The institutional review board at Stanford University exempted this study from review owing to its use of deidentified data. The study followed the STROBE reporting guidelines (eMethods in Supplement 1).

## Results

Excluding 115 162 participants with missing data, the final sample included 398 486 participants. Overall, 27.2% of the sample reported a disability and 2.3% reported high disability (≥4 types). The sample demographics and associated ACE and disability prevalence are shown in Table 1. Compared to participants without a disability, participants with a disability had a higher prevalence and adjusted odds of experiencing any ACE (72.1% vs 60.8%; adjusted odds ratio [AOR], 2.0; 95% CI, 1.9-2.1) and 4 or more ACEs (26.8% vs 14.7%; AOR, 2.6; 95% CI, 2.5-2.8). The prevalence of any ACE was even higher for those with high disability (77.9%; AOR, 2.8; 95% CI, 2.4-3.3), with 36.6% experiencing 4 or more ACEs (AOR, 4.7; 95% CI, 4.1-5.4). A similar pattern was found for individual ACE categories. The prevalence of ACEs and associated AORs were higher across all disability types compared to no disability, with those reporting cognitive impairments having the highest ACE prevalence, followed by independent living limitations (Table 2).

**Table 1.** Demographic Characteristics of the Sample and Prevalence of Adverse Childhood Experiences (ACEs) and Disability^a^

Characteristic	Raw No. (weighted %)	Weighted % (95% CI)		
		Any ACE	≥4 ACEs	Any disability
Overall sample	398 486 (100)	63.9 (63.3-64.4)	18.0 (17.5-18.5)	27.2 (26.6-27.7)
Age, y				
18-29	39 536 (19.1)	71.5 (70.1-72.9)	25.0 (23.6-26.4)	21.7 (20.4-23.0)
30-44	67 861 (25.0)	68.8 (67.5-70.1)	22.8 (21.7-23.9)	17.8 (16.9-18.7)
45-54	57 253 (15.6)	65.9 (64.5-67.3)	19.5 (18.3-20.8)	23.1 (21.9-24.3)
55-64	78 607 (17.1)	63.2 (62.0-64.5)	15.2 (14.3-16.0)	32.0 (30.7-33.2)
65-74	89 055 (13.8)	55.1 (53.7-56.4)	9.9 (9.1-10.6)	34.7 (33.4-36.0)
≥75	66 174 (9.5)	46.0 (44.1-47.8)	5.8 (4.9-6.7)	49.8 (47.9-51.6)
Sex				
Female	218 809 (51.7)	64.1 (63.3-64.9)	19.9 (19.3-20.6)	28.6 (27.9-29.3)
Male	179 677 (48.3)	63.6 (62.8-64.5)	16.0 (15.3-16.7)	25.6 (24.8-26.3)
Race and ethnicity				
American Indian or Alaska Native	6776 (1.0)	75.6 (72.7-78.6)	32.3 (27.3-37.3)	41.1 (37.2-44.9)
Asian	6821 (5.4)	49.4 (44.7-54.2)	9.2 (6.5-11.9)	15.8 (11.7-19.8)
Black or African American	33 157 (10.9)	70.2 (68.9-71.6)	18.1 (17.0-19.3)	28.2 (26.9-29.5)
Hispanic	24 951 (17.7)	64.7 (62.8-66.7)	18.8 (17.2-20.3)	27.4 (25.6-29.2)
Native Hawaiian or Pacific Islander	1037 (0.2)	68.2 (59.4-76.9)	29.2 (18.9-39.6)	22.9 (17.1-28.7)
White	315 373 (62.7)	63.2 (62.6-63.7)	17.9 (17.3-18.4)	27.4 (26.9-27.9)
Multiracial	7876 (1.6)	77.8 (74.0-81.6)	32.0 (28.1-35.9)	34.8 (30.6-38.9)
Other race^b^	2495 (0.5)	67.7 (61.5-73.9)	22.6 (16.5-28.7)	34.6 (28.0-41.2)

^a^ Data are from Washington, DC, and the following states: Alabama, Arizona, Arkansas, California, Delaware, Florida, Georgia, Hawaii, Idaho, Indiana, Iowa, Kansas, Kentucky, Maine, Maryland, Massachusetts, Michigan, Mississippi, Missouri, Montana, Nevada, New Hampshire, New Jersey, New Mexico, New York, North Dakota, Ohio, Oklahoma, Oregon, Pennsylvania, Rhode Island, South Carolina, South Dakota, Texas, Utah, Virginia, West Virginia, Wisconsin, and Wyoming.

^b^ The other race category was only provided with Behavioral Risk Factor Surveillance System data from 2019 through 2021 and includes participants who did not self-select any of the other race categories and were coded as other.

**Table 2.** Prevalence and Adjusted Odds Ratios (AORs) of Adverse Childhood Experiences (ACEs) by Disability Status and Type^a^^,^^b^

ACE category	No disability (n = 273 463)	Any disability (n = 125 023)		High disability (≥4 ACES) (n = 10 493)		Mobility (n = 68 978)		Self-care (n = 16 654)		Independent living (n = 30 768)		Hearing (n = 37 976)		Vision (n = 21 228)		Cognitive (n = 43 806)	
	wt %	wt %	AOR (95% CI)	wt %	AOR (95% CI)	wt %	AOR (95% CI)	wt %	AOR (95% CI)	wt %	AOR (95% CI)	wt %	AOR (95% CI)	wt %	AOR (95% CI)	wt %	AOR (95% CI)
Any ACE	60.8	72.1	2.0 (1.9-2.1)	77.9	2.8 (2.4-3.3)	68.6	1.7 (1.5-1.8)	71.1	1.6 (1.4-1.9)	76.1	2.0 (1.8-2.2)	65.4	1.4 (1.3-1.6)	71.1	1.6 (1.4-1.8)	82.4	2.8 (2.5-3.0)
≥4 ACEs	14.7	26.8	2.6 (2.5-2.8)	36.6	4.7 (4.1-5.4)	22.7	2.1 (1.9-2.3)	29.5	2.5 (2.3-2.8)	35.6	3.0 (2.7-3.3)	19.5	1.7 (1.5-1.9)	27.1	2.1 (1.9-2.3)	40.1	3.5 (3.2-3.8)
Sexual abuse	10.6	20.5	2.3 (2.1-2.5)	32.0	4.3 (3.7-5.0)	19.8	1.9 (1.7-2.1)	25.1	2.4 (2.1-2.8)	28.3	2.6 (2.4-2.9)	16.0	1.6 (1.4-1.8)	21.7	1.9 (1.7-2.1)	28.7	3.0 (2.7-3.3)
Physical abuse	23.0	34.8	1.9 (1.8-2.0)	41.4	2.6 (2.3-2.9)	32.9	1.6 (1.5-1.8)	36.7	1.7 (1.6-1.9)	39.7	2.0 (1.9-2.2)	30.2	1.4 (1.3-1.5)	34.8	1.6 (1.4-1.7)	43.9	2.4 (2.3-2.7)
Emotional abuse	26.8	36.7	1.9 (1.8-2.0)	45.3	3.0 (2.6-3.4)	32.0	1.6 (1.5-1.7)	37.6	1.8 (1.6-2.0)	43.5	2.1 (1.9-2.3)	30.3	1.4 (1.3-1.5)	35.0	1.6 (1.4-1.7)	49.6	2.6 (2.4-2.8)
Domestic violence^c^	15.3	23.7	1.9 (1.8-2.0)	30.9	2.8 (2.5-3.2)	22.2	1.7 (1.5-1.9)	25.3	1.8 (1.6-2.0)	27.5	1.9 (1.7-2.1)	20.3	1.5 (1.4-1.7)	26.5	1.8 (1.6-2.0)	31.0	2.3 (2.1-2.5)
Mental illness^c^	15.8	25.0	2.3 (2.2-2.5)	31.8	3.9 (3.4-4.5)	19.4	1.8 (1.6-1.9)	24.6	2.1 (1.9-2.3)	34.1	2.8 (2.6-3.1)	17.5	1.6 (1.4-1.7)	22.0	1.7 (1.5-1.9)	38.7	3.3 (3.0-3.5)
Substance misuse^c^	24.2	35.2	1.9 (1.8-2.0)	42.6	2.7 (2.4-3.1)	32.4	1.6 (1.5-1.7)	36.2	1.7 (1.6-1.9)	40.4	1.9 (1.8-2.1)	31.3	1.5 (1.4-1.6)	36.2	1.7 (1.6-1.9)	43.7	2.2 (2.0-2.4)
Prison^c^	7.8	12.6	2.2 (2.0-2.4)	17.5	4.0 (3.3-4.8)	9.9	2.0 (1.8-2.2)	12.8	2.2 (1.8-2.5)	16.8	2.5 (2.2-2.8)	8.8	1.7 (1.5-2.0)	13.5	2.0 (1.8-2.3)	18.8	2.4 (2.2-2.7)
Divorce/separation^c^	28.3	33.1	1.5 (1.4-1.6)	37.6	2.0 (1.7-2.3)	29.9	1.5 (1.4-1.6)	33.7	1.5 (1.3-1.7)	38.7	1.7 (1.5-1.8)	25.6	1.2 (1.1-1.3)	33.2	1.4 (1.3-1.5)	41.7	1.7 (1.5-1.8)

Abbreviation: wt, weighted.

^a^ Adjusted for age, sex, and race and ethnicity.

^b^ No disability is the reference for any disability and high disability. The reference for a limitation/impairment AOR is not having the specific limitation/impairment.

^c^ Refers to parent or household member.

## Discussion

This cross-sectional study is, to our knowledge, the largest population-based study reporting the prevalence of ACE exposure in people with disabilities. The data demonstrate that people with disabilities have a high ACE burden compared to those with no disability. Study limitations include the disability questions potentially missing individuals with self-identified disabilities, particularly those with disabilities related to chronic disease and mental health^5^; the retrospective reporting of ACEs; the potential lack of generalizability to other US states; and the inability to fully explore the effect of intersecting identities on ACE burden in people with disabilities. Although research has demonstrated that ACEs impact human health and biological functioning, more research is needed to understand how ACEs affect other aspects of disability (ie, activity and participation limitations, contextual factors) and how ACEs affect the patient care experiences/outcomes for people with disabilities. Addressing ACE exposures in people with disabilities through trauma- and resilience-informed care^6^ should be part of broader efforts to strengthen therapeutic alliances in this marginalized population, with the goals of improving care and health outcomes and reducing health disparities.

## Notes

Supplement 1.
eMethods
**eTable 1.** Disability Questions **eTable 2.** Adverse Childhood Experiences (ACE) Questions, Categories and Criteria
eReferences

Supplement 2. Data Sharing Statement

## References

[1] Soares S, Rocha V, Kelly-Irving M, Stringhini S, Fraga S. Adverse childhood events and health biomarkers: a systematic review. Front Public Health. 2021;9:649825. doi:10.3389/fpubh.2021.649825 34490175 PMC8417002

[2] Grummitt LR, Kreski NT, Kim SG, Platt J, Keyes KM, McLaughlin KA. Association of childhood adversity with morbidity and mortality in US adults: a systematic review. JAMA Pediatr. 2021;175(12):1269-1278. doi:10.1001/jamapediatrics.2021.2320 34605870 PMC9059254

[3] Reynolds JM. National Institutes of Health designates disabled people a health disparity population. JAMA Health Forum. 2024;5(6):e241185. doi:10.1001/jamahealthforum.2024.1185 38874959

[4] Schüssler-Fiorenza Rose SM, Eslinger JG, Zimmerman L, . Adverse childhood experiences, support, and the perception of ability to work in adults with disability. PLoS One. 2016;11(7):e0157726. doi:10.1371/journal.pone.0157726 27379796 PMC4933396

[5] Hall JP, Kurth NK, Ipsen C, Myers A, Goddard K. Comparing measures of functional difficulty with self-identified disability: implications for health policy. Health Aff (Millwood). 2022;41(10):1433-1441. doi:10.1377/hlthaff.2022.00395 36190890 PMC10353341

[6] Ranjbar N, Erb M. Adverse childhood experiences and trauma-informed care in rehabilitation clinical practice. Arch Rehabil Res Clin Transl. 2019;1(1-2):100003. doi:10.1016/j.arrct.2019.100003 33543043 PMC7853397

