# Supplementary material for: Prevalence of Adverse Childhood Experience Exposure by Disability Status
Source: JAMA Health Forum. 2025 Jan 10;6(1):e244881. doi: 10.1001/jamahealthforum.2024.4881 (PMC11724342; doi:10.1001/jamahealthforum.2024.4881)
Supplement: Supplement 1. — eMethods eTable 1. Disability Questions eTable 2. Adverse Childhood Experiences (ACE) Questions, Categories and Criteria eReferences [file jamahealthforum-e244881-s001.pdf]

Supplemental Online Content

Schüssler-Fiorenza Rose SM, Rehkopf DH, Snyder MP, Slavich GM. Prevalence of adverse childhood experience exposure by disability status. *JAMA Health Forum*. 2025;6(1):e244881. doi:10.1001/jamahealthforum.2024.4881

eMethods

eTable 1. Disability Questions

eTable 2. Adverse Childhood Experiences (ACE) Questions, Categories and Criteria

eReferences

This supplemental material has been provided by the authors to give readers additional information about their work.

© 2025 Schüssler-Fiorenza Rose SM et al. *JAMA Health Forum*.

eMethods

The median survey cooperation rate was 73.7% (range: 63.5%-95%) and the median response rate was 47.5% (range: 34.5%-67.2%). We included participants with complete data, excluding 115 162 participants with missing data [Missing: ACE (n = 108,284), disability (n = 31,035), race/ethnicity (n = 1)], yielding a final sample of 398 486 participants.

eTable 1. Disability Questions

**Hearing**	Are you deaf or do you have serious difficulty hearing?
**Vision**	Are you blind or do you have serious difficulty seeing, even when wearing glasses?
**Cognition**	Because of a physical, mental, or emotional condition, do you have serious difficulty concentrating, remembering, or making decisions?
**Mobility**	Do you have serious difficulty walking or climbing stairs?
**Self-Care**	Do you have difficulty dressing or bathing?
**Independent Living**	Because of a physical, mental, or emotional condition, do you have difficulty doing errands alone such as visiting a doctor's office or shopping?

The disability questions (eTable1) are yes/no questions and were coded according to the Centers for Disease control and Prevention (CDC) guide to the disability questions[1].

© 2025 Schüssler-Fiorenza Rose SM et al. *JAMA Health Forum*.

eTable 2. Adverse Childhood Experiences (ACE) Questions, Categories and Criteria

I. Sexual Abuse Category (criterion: once or more than once to any of following 3 questions)

How often did anyone at least 5 years older than you or an adult...

1) Ever touch you Sexually?

2) Try to make you touch them ?

3) Force you to have sex?

III. Physical Abuse Category (criterion: once or more than once)

4) Before age 18, how often did a parent or adult in your home ever hit, beat, kick or physically hurt you in any way? Do not include spanking.

II. Emotional Abuse Category (criterion: more than once)

5) How often did a parent or adult in your home ever swear at you, insult you, or put you down?

IV. Domestic Violence Category (criterion: once or more than once)

6) How often did your parents or adults in your home ever slap, hit, kick, punch or beat each other up?

VII. Mental Illness Category (criterion: yes)

7) Did you live with anyone who was depressed, mentally ill or suicidal?

VI. Substance Abuse Category: (criterion yes to 1 or both)

8) Did you live with anyone who was a problem drinker or alcoholic?

9) Did you live with anyone who used illegal street drugs or who abused prescription medication?

V. Incarcerated Household Member (criterion: yes)

10) Did you live with anyone who served time or was sentenced to serve time in a prison, jail, or other correctional facility?

VIII. Divorce Category (criterion: yes)

11) Were your parents separated or divorced?

The Adverse Childhood Experiences questions (eTable2) include 11 questions that ask about experiences that occurred before the age of 18[2]. These 11 questions were coded and combined into 8 categories following the method described by Ford et al.[3] including coding answers of "don't know" as negative.

The race/ethnicity data are self-reported from participants. The BRFSS provides a variable used for internet prevalence tables which imputes race/ethnicity of those who refuse to answer by using the most common race/ethnicity response for that region of the state. An other category was included in 2019-2021 but not in 2022 data and represents people who did not choose any of the offered race categories.

© 2025 Schüssler-Fiorenza Rose SM et al. *JAMA Health Forum*.

eReferences

1. Centers for Disease Control and Prevention National Center on Birth Defects and Developmental Disabilities. A Data Users' Guide to the Disability Questions Included in the Behavioral Risk Factor Surveillance System. Centers for Disease Control and Prevention; 2018. https://www.cdc.gov/brfss/data_documentation/pdf/BRFSS_Data_Users_Guide_on_Disability_Questions_2018-508.pdf

2. Centers for Disease Control and Prevention. Behavioral Risk Factor Surveillance System Survey Questionnaire. US Department of Health and Human Services, Centers for Disease Control and Prevention; 2019, 2020, 2021 2022.

3. Ford ES, Anda RF, Edwards VJ, et al. Adverse childhood experiences and smoking status in five states. Prev Med. 2011;53:188-193.

© 2025 Schüssler-Fiorenza Rose SM et al. *JAMA Health Forum*.
