# Supplementary material for: Prevalence of Adverse Childhood Experience Exposure by Disability Status
Source: JAMA Health Forum. 2025 Jan 10;6(1):e244881. doi: 10.1001/jamahealthforum.2024.4881 (PMC11724342; doi:10.1001/jamahealthforum.2024.4881)
Supplement: Supplement 2. — Data Sharing Statement [file jamahealthforum-e244881-s002.pdf]

Data Sharing Statement

Schüssler-Fiorenza Rose. Prevalence of Adverse Childhood Experience Exposure by Disability Status. *JAMA Health Forum*. Published January 10, 2025.
doi:10.1001/jamahealthforum.2024.4881

Data
Data available: Yes
Data types: Deidentified participant data, Data dictionary
How to access data: All data used in this study are available from the CDC BRFSS website: https://www.cdc.gov/brfss/annual_data/annual_data.htm
When available: beginning date: 09-15-2023

Supporting Documents
Document types: Statistical/analytic code
How to access documents: Statistical/Analytic code can be obtained from the corresponding author: smrose11@stanford.edu
When available: With publication

Additional Information
Who can access the data: Anyone can access the BRFSS website
Types of analyses: For any purpose consistent with BRFSS guidelines
Mechanisms of data availability: Data can be freely downloaded by going to the BRFSS website.
